# Is the Self Always Better than a Friend? Self-Face Recognition in Christians and Atheists

**DOI:** 10.1371/journal.pone.0037824

**Published:** 2012-05-25

**Authors:** Yina Ma, Shihui Han

**Affiliations:** Department of Psychology, Peking University, Beijing, People’s Republic of China; University of Western Brittany, France

## Abstract

Early behavioral studies found that human adults responded faster to their own faces than faces of familiar others or strangers, a finding referred to as self-face advantage. Recent research suggests that the self-face advantage is mediated by implicit positive association with the self and is influenced by sociocultural experience. The current study investigated whether and how Christian belief and practice affect the processing of self-face in a Chinese population. Christian and Atheist participants were recruited for an implicit association test (IAT) in Experiment 1 and a face-owner identification task in Experiment 2. Experiment 1 found that atheists responded faster to self-face when it shared the same response key with positive compared to negative trait adjectives. This IAT effect, however, was significantly reduced in Christians. Experiment 2 found that atheists responded faster to self-face compared to a friend’s face, but this self-face advantage was significantly reduced in Christians. Hierarchical regression analyses further showed that the IAT effect positively predicted self-face advantage in atheists but not in Christians. Our findings suggest that Christian belief and practice may weaken implicit positive association with the self and thus decrease the advantage of the self over a friend during face recognition in the believers.

## Introduction

To recognize one’s own face in a mirror reflects an ability to distinguish the self from others [1] and has been suggested to be an indicator of self-awareness [2]. Self-face recognition in human adults is characterized by faster responses to self-face than to others’ faces [3], [4]. The self-face advantage in reaction times (RTs) is eliminated when an implicit positive association (IPA) with self-concept is weakened by self-concept threat priming that requires self-reflection on negative personality traits [5], suggesting that the self-face advantage is mediated by an implicit positive association with the self.

Because social psychological research suggests that self-concept is essentially a social construction [6], one may expect that self-face recognition associated with the positive view of the self is influenced by social and cultural experiences. Indeed, a recent study found that self-face advantage in RTs was significantly reduced in Chinese graduate students when responded to self-face and a faculty advisor’s face and the decrease in self-face advantage positively correlated with the degree of fear of negative evaluations from advisors [7]. Interestingly, a following study showed that European American graduate students maintained the self-face advantage when they responded to self-face and a faculty advisor’s face [8], suggesting less social influence on self-face recognition in a Western cultural context. Another cross-cultural study also found that self-face advantage in RTs was greater in British compared to Chinese participants [9]. These results together suggest that cultural experience may interact with social relationship to affect the process involved in self-face recognition. The findings can be understood in the framework that the Western independent self emphasizes autonomous self-identity and results in enhanced attention to the self than to others whereas the East Asian interdependent self emphasizes fundamental social connections and results in sensitivity to information related to significant others [10]. It appears that the cultural difference in self-concept significantly affects social cognitive processes involved in self-face recognition.

The current work further investigated whether and how Christian belief and practice influence self-face recognition in a Chinese population. Shared religious belief and knowledge, referred to as a subjective culture [11], strongly influence human behaviors and thoughts. Christians constitute a minority group of members of the Chinese society and are dominated by Protestant fundamentalism [12]. Christian fundamentalists put a heavy emphasis on human sinfulness [13] and such belief of human nature leads to a negative self-image and a call for denial of the self in Christians [14], [15]. Our recent study has shown that Christian fundamentalists’ belief and practice affect self reflective thoughts of personality traits by weakening encoding of self-relevance of trait words in a self-referential task [16] in Christian compared to Atheist Chinese [17]. However, it remains unclear whether and how Christian belief and practice modulate self-face recognition.

**Figure 1:**
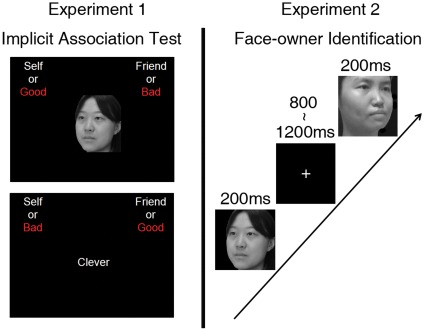
Illustration of the stimuli and procedure in the current study. (a) Illustration of the stimuli and procedure in the IAT in Experiment 1. (b) Illustration of the stimuli and procedure in the face-owner identification task in Experiment 2.

Because Christian fundamentalists’ belief and practice result in negative self-image or denial of the self [13]–[15], we hypothesized that the IPA with self-face in Chinese Christians is weakened relative to that in Chinese atheists. In addition, as self-face advantage reflects positive attitude toward the self [5], weakened IPA with self-face may consequently reduce self-face advantage in Christians. We conducted two experiments to test these hypotheses. Experiment 1 compared the IPA with self-face over a friend’s face in Christian and Atheist participants using the typical implicit association test (IAT, [18]). Experiment 2 assessed self-face advantage over friend-face in the same Christian and Atheist participants by measuring RTs to self-face and friend-face in a face-owner identification task (Figure 1). Hierarchical regression analyses were conducted to further assess whether religious belief and practice affect the relationship between the IPA with self-face and the self-face advantage across individuals. If the IPA mediates the self-face advantage in atheists, we would expect larger self-face advantage in those with greater IPA with self-face. However, we would not expect a positive correlation between the IPA with self-face and the self-face advantage across Christian participants if the IPA with self-face does not underlie the self-face advantage in Christian individuals.

## Methods

### Subjects

Forty Chinese undergraduate and graduate students participated in our study as paid volunteers. Ten pairs of participants were self-identified Christians (10 males and 10 females, 19–27 years) who were members of local faith communities and had been attached to them for 1 to 20 years (mean year ± SD = 4.39±4.76). 95.0% of the Christians reported to attend Church or fellowship at least once a week, to pray every day, and to read the Bible at least once a week. Christian participants’ religious attitude was evaluated using a questionnaire containing 6 religious items derived from Minnesota Multiphasic Personality Inventory such as “I believe there is a God”, “I believe the importance of praying to Jesus”, “I believe the importance of reading the Bible”. A 5-point scale was used to assess their religious attitude with 0 = absolutely disagree, 1 = disagree, 2 = agree to a certain degree, 3 = agree, and 4 = strongly agree. The mean rating score was 3.7±0.2 for Christian participants. Ten pairs of participants were self-identified atheists (10 males and 10 females, 20–27 years) who self-reported not to believe in any religion. The Christian and non-religious participants were matched on educational level (2–7 years university). Each pair of participants were age/gender matched friends and knew each other for at least two years during which they were roommates or classmates. All participants were right-handed, had normal or corrected-to-normal vision, and self-reported no neurological or psychiatric history. This study was approved by the ethics committee at the Department of Psychology, Peking University. All participants gave written informed consent before the study. Two participants gave written informed consent (as outlined in the PLoS consent form) to publication of their photographs in Figure 1.

### Stimuli and Procedure

Ten face images of each participant, with a neutral facial expression, were taken using a digital camera. Participants’ heads were oriented to the left (from 30°to 90°) in five images and to the right in other images. Face stimuli of each pair of participants were used as self-face and friend-face so that perceptual features of faces were identical in self-face and friend-face conditions. All images were calibrated in luminance and contrast, and were converted into JPG format. Each picture stimulus was shown on a 17-inch color monitor and subtended a visual angle of 2.13^o^ × 2.17^o^ (width × height) at a viewing distance of 70 cm.

### Implicit Association Test

Figure 1a illustrates the IAT task used in Experiment 1. Four kinds of stimuli were used in the IAT task, i.e., me items (self-face), not me items (friend-face), positive items (positive trait adjectives) and negative items (negative trait adjectives). There were 7 blocks of categorization trials (see Table 1 for details). Blocks 1, 2, 5 were used to make participants get familiar with the correspondence of responding hand and the category label in order to obtain high response accuracy. Each stimulus was presented for 300 ms at the center of the screen and was followed by a fixation cross with a duration varying between 900 to 1500 ms (mean = 1200 ms). On each trial participants responded to the stimulus by pressing a key on a standard keyboard using the left or right index finger. The IAT effect was measured as the difference in RTs between face stimuli associated with negative vs. positive items, similar to the previous work [18], [19]. The assignment of category labels to the left and right hands were counterbalanced across subjects within each subject group.

**Table 1:** A list of the categorization tasks in IAT in Experiment 1.

Blocks	Category	labels
1 (practice, 20trials)	Self-face items	Friend-face items
2 (practice, 20trials)	Positive items	Negative items
3 (practice, 20trials)	Self-face +Positive items	Friend-face +Negative items
4 (critical, 40trials)	Self-face +Positive items	Friend-face +Negative items
5 (practice, 20trials)	Negative items	Positive items
6 (practice, 20trials)	Self-face +Negative items	Friend-face +Positive items
7 (critical, 40trials)	Self-face +Negative items	Friend-face +Positive items

Note: Seven blocks of categorization trials were conducted for each participant. There were 4 kinds of stimuli in the IAT task, i.e., me items (self-face), not me items (friend-face), positive items (positive trait adjectives) and negative items (negative trait adjectives). On each block participants responded to the stimuli according to the category labels.

### Face-owner Identification Task

Figure 1b illustrates the face-owner identification task used in Experiment 2. Each subject was asked to perform two blocks of trials, responding with the left and right hand, respectively. Each block consisted of 20 self-faces, and 20 friend-faces, which were presented in a random order. On each trial, a stimulus was presented for 200 ms at the center of the screen, followed by the presentation of a fixation cross with a duration varying between 800 to 1200 ms (mean = 1000 ms). Subjects were asked to identify face owners (self vs. friend) by pressing one of the two keys. The assignment of self-face and friend-face responses to the index and middle fingers was counterbalanced across participants. Participants responded with the left hand in one block and with the right hand in another block. The order of the responding hand was also counterbalanced across subjects in each subject group. Instructions emphasized both response speed and accuracy.

### Hierarchical Regression Analysis

To examine whether Subject Group (Atheists vs. Christians) affected the relationship between IPA with self-face (independent variable, IV) and the self-face advantage (dependent variable, DV, calculated by subtracting left hand responses to self-face from those to friend-face), we performed moderated hierarchical regression analysis. To do this, we first normalized the IV (IAT effect from Experiment 1, indexed by mean latency for (self-face + negative) block minus mean latency for (self-face + positive) block) and the covariate variable (Subject Group). The interactions between the IAT effect and Subject Group were calculated by multiplying the normalized variables together [20]. Normalized Subject Group (the moderator), IAT effect (IV), and their interactions were sequentially entered into the moderated hierarchical regression. The moderator effect was indicated by a significant interaction on the self-face advantage observed in Experiment 2. As a significant moderator effect of Subject Group on the IAT/self-face advantage relationship was observed, we also conducted *post hoc* regression analyses for the Atheist and Christian group, respectively.

## Results

RTs with correct responses and within three standard deviations were analyzed and reported. Repeated measures analyses of variance (ANOVAs) were conducted on both response accuracies and RTs. Since response accuracies were high (>90% in both experiments) and ANOVAs of response accuracies did not show any significant effect, only RT results were reported in details.

### Experiment 1: Implicit Association Test

We calculated the IAT effect in the same way as Greenwald et al.’s study [18]. The IAT effect was defined by the difference in mean RTs between (self-face + negative) block and (self-face + positive) block. The IAT effect was significant in atheists (mean RTs in the (self-face + negative) block  = 605±94 ms; mean RTs in the (self-face + positive) block = 553±57 ms, IAT effect = 53±64 ms, t _19_ = 3.652, p = 0.002), suggesting that atheists hold a stronger implicit positive attitude toward the self than toward friends. RTs in Christian participants failed to show a significant IAT effect (mean RTs in the (self-face + negative) block = 616±123 ms; mean RTs in the (self-face + positive) block = 609±128 ms, IAT effect = 6±76 ms, t _19_ = 0.367, p = 0.718), suggesting that Christian participants hold comparable implicit positive attitude toward the self and friends. To confirm the difference in the IAT effect between the two subject groups, we conducted the independent-sample t-test between Christian and Atheists groups, which showed a significant group effect (t _38_ = 2.071, p = 0.045), confirming a significantly reduced implicit positive association with self-face in Christian than Atheist participants.

### Experiment 2: Face-owner Identification Task

Because the previous studies have shown evidence for hand difference in self-face recognition (i.e., stronger self-face advantage with left-hand compared to right hand responses [3], [5], we also analyzed left-hand and right-hand responses separately. RTs were subjected to ANOVA with Face (self-face vs. friend-face) and Hand (Left vs. Right hand) as independent within-subjects variables and Subject Group (Atheists vs. Christians) as a between-subjects variable. There was a significant 3-way interaction of Face x Hand x Group (F_1, 38_ = 5.478, p = 0.025). Post hoc analyses were conducted separately for atheists and Christians, and confirmed that the Face x Hand interaction was only true for atheists (F_1, 19_ = 5.854, p = 0.026) but not for Christian F_1, 19_ = 1.065 p = 0.315), suggesting different self-face advantage between the left and right hand responses in atheists but not in Christians. Post hoc analyses showed that Atheist participants responded faster to self-face compared to friend-face with the left hand responses (F_1, 19_ = 5.088, p = 0.036) but not with the right hand responses (F_1, 19_ = 0.058, p = 0.812, see Table 2 for the RTs in details). However, Christians showed comparable RTs to self-face and friend-face with both the left and right hand responses (ps >0.3).

**Table 2:** Mean RTs(ms) (SD) and difference in RTs in Experiment 2.

Faces	Atheists		Christians	
	Left	Right	Left	Right
Self-face	493 (71)	491 (66)	529 (93)	506 (71)
Friend-face	519 (63)	493 (58)	525 (83)	514 (71)
Difference	27(53)*	2(41)	−4(41)	8(34)

Note: There were twenty participants in each group of participants.

* p<0.05.

### Hierarchical Regression Analyses

**Figure 2:**
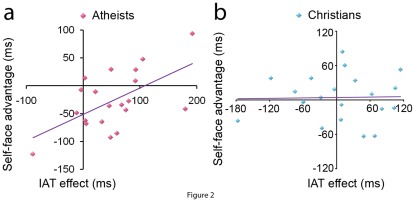
Correlation Result of Atheists and Christians. The X-axis represents the IAT effect (i. e., RTs to self-face when it is associated with negative items minus when associated with positive items). The Y-axis represents self-face advantage (i. e., left hand RTs to self-face minus those to friend-face in the Face-owner identification task).

Hierarchical regression analyses were conducted to examine whether Subject Group (Atheists vs. Christians) affected the relationship between the IPA with self-face (IV) and the self-face advantage (DV). The model regressed the moderator, IV (normalized IAT effect), and their interaction. This analysis showed that the interaction of Subject Group and the IAT effect was predictive of individuals’ self-face advantage (F = 4.949, p = 0.006; see Table 3 for statistic details), suggesting that the IAT effect predicted one’s self-face advantage differently between Atheist and Christian participants. Post hoc regression analyses confirmed a positive correlation between the IAT effect and the self-face advantage in Atheist participants (β = 0.583, p = 0.007, Figure 2a) but not in Christian participants (β = 0.022, p = 0.927, Figure 2b). These results suggested that greater IPA with self-face positively predicted larger self-face advantage (i.e., faster responses to self-face than to friend-face) in Atheist participants but not in Christian participants.

**Table 3:** Hierarchical Regression Analysis on IAT effect with the self-face advantage as the Dependent Variable.

	Step1 β	Step2 β
IAT effect	0.307	0.018
Group	−0.416*	−0.435**
IAT x Group		0.440*
ΔR^2^	0.186	0.106
ΔF	4.229*	5.386*
R^2^	0.168	0.292
Adjusted R^2^	0.142	0.233
Overall F	4.229*	4.949**
Df	37	36

* p<0.05,

** p<0.01.

## Discussion

Previous research suggests that Christian belief and practice that emphasize human sinfulness [13] may weaken positive attitude toward the self [14], [15] and reduce neural encoding of self-relatedness of personality trait words [17]. In two experiments the current work tested the hypothesis that the influence of Christian belief and practice on self-related processing may extend into the perceptual domain by reducing the implicit positive association with self-face and weakening the self-face advantage during face recognition. Experiment 1 found that, while Atheist participants responded faster to self-face when it was associated with positive than with negative trait words, this IAT effect was significantly reduced in Christian participants. Experiment 2 found that Atheist participants responded faster to self-face compared to friend-face, replicating the robust self-face advantage [3]–[5]. However, the self-face advantage was significantly weaker in Christian than in Atheist participants. Furthermore, the hierarchical regression analysis showed that the relationship between the IAT effect and the self-face advantage also differed significantly between Atheist and Christian participants, with a positive correlation between the IAT effect and the self-face advantage in atheists but not in Christians.

The results in Experiment 1 support our first hypothesis that the implicit positive attitude toward self-face is weakened in Christian relative to Atheist participants. According to the IPA theory of self-face advantage [5], the implicit positive attitude toward the self plays a pivotal role in the self-face advantage in behavioral responses during face recognition. Thus given the IPA theory and the results of Experiment 1, it can be assumed that the decreased self-face advantage in Christian than Atheist participants arose from the weakened IPA with self-face.

The results of hierarchical regression analyses further support the association between the IPA with self-face and self-face advantage in Atheist participants but not in Christian participants. Thus our findings on the one hand support the IPA theory by showing evidence for the association between the implicit positive view of the self and the self-face advantage. On the other hand, our results suggest that the implicit positive view of the self can be reduced by Christian belief and practice that repudiates the distinctness of the self and friends and this in turn can eliminate the advantage of self-face over friend-face in the believers.

Previous studies have shown evidence that Christian belief and practice influence social cognitive processes [17], [21]–[23]. For example, it has been shown that Christian belief and practice decreased self-relevance encoding during self-reflection [17], and increased prosocial behaviors [21] and implicit self-regulation [22]. Priming Christian religious concepts also led to increased racial prejudice [23]. Our work compliment previous work by showing that Christian belief and practice also affect self-related processing in the perceptual domain by adopting a weakened positive association with self-concept advocated by Christianity. Similarly, the difference in self-concept between Western and East Asian cultures also gives rise to the variation of self-face advantage across Westerners and Chinese [5], [8]. A recent event-related brain potential study showed evidence for a greater self-face advantage in RTs in British than in Chinese participants [9]. Cultural difference also exists in the neural mechanisms underlying self-face recognition. Relative to friend-face, self-face elicited an enhanced frontal activity at about 200 ms after stimulus onset in Westerners, whereas a reverse pattern was observed in Chinese. Thus an unresolved issue related to the current work is whether the neural mechanisms underlying self-face recognition are different between Christian and atheists. This can be examined in future work that combines brain imaging and the self-face recognition paradigm used in the current study.

There are several limitations in the current study. First, the current work tested the difference in self-face recognition between Christian and Atheist participants in a specific sociocultural context (i.e., Chinese culture). Christians constitute a minority group of members of the current society in China [12] and this is different from the situation in the Western societies. Thus it is unclear whether Christian fundamentalism in the Western societies may influence self-face recognition in a similar vein. Further research may test Christian participants in Western cultures in order to examine whether Christian belief and practice produce similar influence on self-face recognition in different sociocultural environments.

Second, there has been evidence that self-construals influence the neural representation of the self and close others. It has been shown that, relative to priming Western cultures, priming East Asian cultures led to similar neural representation of personality traits of the self and a close other in the medial prefrontal cortex [24]. Moreover, relative to interdependent self-construal priming, independent self-construal priming increased the right frontal activity that differentiated self-face from faces of familiar others [25]. Because there has been no research report of cultural values and self-construals of Chinese atheists and Christians and these were not measured either in the current work, it is unknown to what degree our Atheist and Christian participants were different in self-construals and whether the difference in self-construals, if any, may contribute to the difference in self-face recognition in the two subject groups. One of our recent studies measured self-construals using the Self-construal Scale [26] and the pilot data suggest that both Christian and Atheist participants exhibited greater interdependent than independent self-construal scores [Ma and Han, unpublished data]. Future research should clarify how self-construals contribute to the difference in self-face advantage between atheists and Christians.

Finally, although the behavioral performances in the face-own identification task suggested a different relation between self and a close other in Atheist and Christian participants, the current work did not measure subjective feelings of self-friend relationship and thus was unable to address how the relationship between the self and a friend influences self-face recognition in the two subject groups. The current work only tested a small number of participants. Future work may test whether the conclusion based on our findings can be applied to a large population.
